# The Construction of Unsmooth Pulse Images in Traditional Chinese Medicine Based on Wave Intensity Technology

**DOI:** 10.1155/2016/2468254

**Published:** 2016-11-24

**Authors:** Jie-wei Luo, Si-wei Guo, Shuang-shuang Cao, Ning Lin, Zhen-sheng Ye, Shi-chao Wei, Xing-yu Zheng, Miao-miao Guo, Xiao-rong Meng, Fang-meng Huang

**Affiliations:** ^1^Provincial Clinical Medical College, Fujian Medical University, Fuzhou 350001, China; ^2^Department of Traditional Chinese Medicine, Fujian Provincial Hospital, Fuzhou 350001, China; ^3^Fujian University of Traditional Chinese Medicine, Fuzhou 350108, China

## Abstract

Unsmooth pulse is one of the most important pulses in TCM diagnostics. We constructed the wave intensity (WI) images of unsmooth pulse based on the pressure wave (*P*), flow velocity wave (*U*), and WI [(*dP*/*dt*)(*dU*/*dt*)] by ALOKA Prosound *α* 10 Color Doppler. The characteristic of Cunkou normal pulse could be summarized as follows: compared to Renying pulse, its* W*1 amplitude is smaller and NA wave is more obvious, while the* W*2 wave is indistinct or even invisible, and the* R*-1st is longer than that of Renying pulse. The principal* U* wave of Renying pulse looks like “Λ” shape, while it looks like an arched blunt “*∩*” shape in Cunkou pulse, and the amplitude of* U* wave in Cunkou pulse is smaller. The direction of the principal* U* wave in Cunkou unsmooth pulse is up, which shows hoof boots “h” shape with high amplitude and a significant notch on declined branch; the amplitude of predicrotic wave in unsmooth pulse* P* wave is significantly higher, which could be even higher than that of h1, resulting in early appearance of h3 or integrating with h1, which forms a wide and blunt peak. Unsmooth pulse shows poorer vascular elasticity and greater vascular stiffness.

## 1. Introduction

Wave intensity (WI) is a state-of-the-art technology applied in the study of cardiovascular hemodynamics and its correlation with the heart and vessels [1]. It is based on the echo tracking (ET) by the Japanese Aloka Company. The English scholars Parker and Jones were the first to suggest developing a real-time [2], noninvasive wave intensity analyzing system via integrating the technology of color Doppler and echo tracking (ET). Wave intensity (WI) is defined as the product of *dP*/*dt* and *dP*/*dt* (WI = (*dP*/*dt*)(*dU*/*dt*)), where *dP*/*dt* and *dU*/*dt* are, respectively, the change of pressure and velocity during a sample time, measured simultaneously at the same site [3]. In addition to cardiovascular system, WI has a wide range of application in other field of system [1, 4, 5].

Pulse condition, which has been identified as a crucial basis of Syndrome Differentiation and Disease Differentiation, is one of the most significant types of guidance of treatment in Traditional Chinese Medicine (TCM). In TCM, pulse condition refers to the frequency of pulse, pulse strength, and the place where a pulse can be taken, which reflects the image and the dynamics of the pulse, serving as the most significant reference of the Syndrome Differentiation of TCM. In Jin Dynasty, Wang Shuhe firstly summed up the twenty-four pulses in his book* Mai Jing (Pulse Classic)*, and it was until Ming Dynasty that Li Zhongzi finally identified that there were 28 kinds of pulse conditions in his book* Zhen Jia Zheng Yan (Key to Physicians)*. Different pulse condition reflects the functional diversification of five* Zang*-Viscera, six* Fu*-Viscera, four limbs, skeleton, five sense organs, nine orifices, skin, muscles, tendons, meridian, vessel, and so forth. For example, as one of the most important pulses, the clinical significance of unsmooth pulse is lack of essence and insufficiency of blood,* qi* stagnation and blood stasis, and food-phlegm retention. It is believed that unsmooth pulse are more common in the elderly and patients with cardiovascular system damage or systemic disease, which not only reflects the disorder of hemodynamics and blood rheology but also reflects an increase of vessel stiffness and decreased elasticity. Our traditional pulse examination, however, has limited development since it could only be felt but could not be delivered.

To date, it is delightful that WI technology has made the comprehensive analysis of the three-dimensional dynamic movement of blood vessels possible via simultaneously recording the blood flow velocity, pressure, wall movement, the change of the diameter of the blood vessels, and so forth [6], aiming at accurately showing the basically four elements of pulse condition, namely, the location, rate, shape, and power in traditional pulse conditions, which was used to explain and classify the features of the 28 pulses. In modern times, with the further understanding and research related to sphygmology literature, we learned a lot about pulse condition from eight aspects, namely, pulse location, number, length, strength, width, smooth degree, tense degree, and even degree [7]. The previous studies of pulse image were based on baroreceptor which stated the two-dimensional status of pressure and time. Different pressure on the probe according to different operators along with different thickness of skin and soft tissue could easily lead to distortion of the sphygmograms and described only one side of the pulse image. The application of WI technology overcomes all these defects, combines the pressure wave with the wave flow, and can reflect a more realistic, comprehensive, and three-dimensional status of blood flow and pulse overall.

In recent years, studies discovered that the vascular pressure indicated numerous quantities of information of diseases whether it is close to cardiovascular or peripheral blood vessels, which drove researchers to devote more enthusiasm to this field. Study on the diagnosis of TCM pulse was as significant as cardiovascular functional research. These two studies can supplement each other and better reveal the Chinese Medicine theory from mutual learning and common development. At present, the invention of noninvasive method to estimate the central artery pressure has been really successful. SphygmoCor pulse wave analyzer, A-PULSECASPro, and BPro™ collect the radial artery (*Cunkou pulse*) according to the surface pressure* via* Hi-Fi probe and obtain the pressure waveform of the central artery (aortic root) by mathematical transformation. After adjustment of the brachial artery systolic pressure and diastolic blood pressure, they calculate the central artery pressure [8–10]. The position of central artery pressure is quite similar to the Cunkou pulse theory of Chinese medicine which considers the understanding of Cunkou pulse can determine the function of organs. Therefore, with the development of imaging technology, similar to the noninvasive measurement of arterial pressure and vascular elasticity, the technology of pulse image has a bright prospect for clinical application.

The author constructed the pulse condition image of unsmooth pulse based on the WI technology so that the pulse images could be more intuitive and recognizable, which could enrich the objective research of Chinese medicine. The application of WI technology in describing the pulse condition is intended to shed light on the bright prospect of the diagnostics of the Traditional Chinese Medicine.

## 2. Materials and Method

### 2.1. Patients

Thirty patients with pulse diagnosed as unsmooth pulse between July 2014 and April 2015 who were examined at the Fujian Provincial Hospital inpatient or outpatient were enrolled on our study, including 18 males and 12 females, whose average ± SD age was (62.00 ± 4.95); we also include 30 healthy patients who were diagnosed as normal pulse at the Fujian Provincial Hospital outpatient or Fujian University of Traditional Chinese Medicine with 15 males and 15 females, and their average ± SD age was (33.13 ± 7.67). There was no statistical gender difference between the 2 groups (*p* > 0.05) and the diagnosis of pulse condition was performed by five experienced deputy or chief Traditional Chinese Medicine physicians according to a fixed order. They contributed equally to the final result. This study was approved by the Medical Ethics Committee of Fujian Provincial Hospital. All participants gave signed informed consent and were handled according to the Ethics Committee Regulatory Guidance.

Inclusion criteria refer to the features of unsmooth pulse [7], which is thready, slow, and short, with unsmooth coming and going, like scraping a piece of bamboo. Characteristics of normal pulse, for healthy people, is that pulse can be felt at the* cun*,* guan*, and* chi* portions, having a frequency of about four to five beats per breath, neither floating nor deep, and neither big nor small. It is even, stable, harmonious, and forceful with regular rhythms. When a heavy pressure is applied to the* chi* pulse, one may feel a forceful beating. It varies with physiological and climatic conditions. Ancient people concluded that a normal pulse should reflect existence of adequate stomach* qi*, existence of vitality, and existence of kidney* qi*. Since unsmooth pulse could be slow or rapid with irregular or regular intermittence, patients of unsmooth pulse along with arrhythmic or thready and forceless pulse were excluded in this study.

The sample size for the comparison of sample mean was calculated according to *n*_1_ = *n*_2_ = 2[(*t*_*α*_ + *t*_2*β*_)*S*/*δ*]^2^, *n*_1_ = *n*_2_ represents the size of the two samples, the variance is *ν* = *n*_1_ + *n*_2_ − 2, *t*_*α*_ and *t*_2*β*_ mean the *t* value of *α* and 2*β* at statistical significance level, *S* refers to the standard deviation, and *δ* refers to a difference according to the researcher's request. Regarding the level of *α* = 0.05 and *β* = 0.1, the minimum sample size required for each WI index is considered; finally the minimum sample size was determined as *n*_1_ = *n*_2_ = 28.

### 2.2. Apparatus

In this study, Aloka Prosound *α* 10 (SN 200N8431) Color Doppler Ultrasound detector was applied with a 1–5 MHz heart broadband probe and a 5–13 MHz vascular probe (Hitachi Aloka Medical, Ltd., Tokyo, Japan). All participants were informed to have the test at least 1 hour after their meals at the temperature of 20–24°C.

### 2.3. Blood Pressure Test

The mercury sphygmomanometer was used to measure the blood pressure of right upper extremity and all the subjects were examined at the supine position after 10-minute rest. Take the average pressure of the two tests as the final result.

### 2.4. The WI Detection of Cunkou Pulse [11–13]

**Figure 1:**
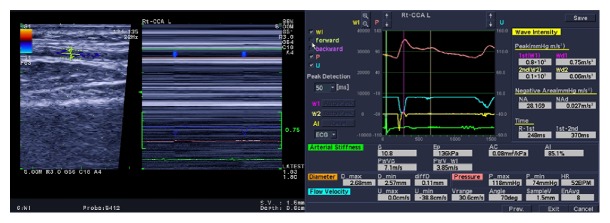
Collection screen and result screen of WI. Pressure wave (*P*) curve is marked red, blood flow velocity wave (*U*) curve is marked blue, WI (*dP*/*dt*)(*dU*/*dt*) curve is marked yellow, and curve of electrocardiogram wave is marked green.

The subject was instructed to rest for at least 10 minutes before WI examination in a quiet condition at the temperature of 20–24°C. Coffee or strong tea was strictly forbidden before the examination. Subjects were examined at the supine position, and the blood pressure of right upper limb of each participant was measured twice. The EKG was connected at the same time and selected the right radial artery as the inspected artery. The right arm was supposed to be mildly abducted, with palm up and muscle relaxed. The analysis of WI was set up as follows: set the angle of “Beam Steer (B)” of the WI at 15°, “Beam Steer (Flow)” at −15°, and “Angle correct” at 60°. Take the long axis of the vessel as the tangent plane and moderately adjust the probe to let the detected vessel come in view obliquely, while ensuring the angle between blood flow and the direction of Doppler acoustic beam was less than or equal to 60° with the two-dimensional sampling gate (width: 2.5 mm) perpendicular to the arterial wall. The WI mode was initiated after clicking “WI” on the LCD under the B/M model. Modify the Cunkou normal pulse (radial artery) of color Doppler, a minimal value without the appearance of blood flow aliasing. Press the button of “Select” and start to collect images and data. The tracing waves should be at least six, and more than five waves were required to be analyzed. After affirming that the WI images meet the requirement, press the “Freeze” button to finish image collection. Shift the tracing ball and review and select a few cardiac cycle images with relative consistent alternative curve of vessel diameter. Then press “Select” button to replay the two-dimensional images and select images with clear demonstration of the adventitia and media of the vascular wall and well displayed color Doppler images, and press the “Store” button and save the first image; enter the average result of blood pressure; thereafter initiate the analyzing step. Finally four images were saved for further analyzing and more details of WI were shown in Figure 1.

### 2.5. Main Parameters of the WI Detection [12, 14]

Wave intensity (WI) is defined as the product of *dP*/*dt* and *dP*/*dt* (WI = (*dP*/*dt*)(*dU*/*dt*)), where *dP*/*dt* and *dU*/*dt* are, respectively, the change of pressure and velocity during a sample time, measured simultaneously at the same site. It is obvious that the waveform of WI is formed by the product of the two changing phases, namely, the pressure and velocity (*dP*/*dt*, *dU*/*dt*). The waveform of WI consists of *W*1, *W*2, and NA. The forward traveling compression wave (*W*1) illustrates that the change of positive pressure accelerates blood flow: the forward traveling expansion wave (*W*2) indicates the reduced pressure leading to the descending of blood flow and the closure of aortic valve and the negative area formed by the backward traveling compression wave (NA) shows that the reduction of velocity is resulting from the pressure augmentation. The major parameters of WI consist of *W*1, *W*2, NA, *R*-1st, and 1st-2nd. *W*1 appears in the early contraction, which reflects the left ventricular systolic function, and it is closely related to the velocity of blood flow at the beginning of ejection. NA appears in the middle of contraction, and it is due to increased pressure and decreased blood flow rate; *W*2 appears during the end of systole and it exhibits left ventricular function between late systole and isovolumetric diastolic phase, which is correlated to the decreased pressure, slowed down velocity of blood flow, and the closure of aortic valve [13]. Parameters of time are *R*-1st and 1st-2nd. *R*-1st (predejection period) is the interval between the peak of the R wave and the peak of the *W*1 wave; 1st-2nd (ejection period) is the interval between *W*1 peak and *W*2 peak. Parameters of arteriosclerosis are *β*, *E*_*p*_, AC, AI, PWV*β*, and PWV-WI. Vascular Stiffness (*β*) = In⁡(*P*_*s*_/*P*_*d*_)/[(*D*_*s*_ − *D*_*d*_) − *D*_*d*_], elastic modulus (*E*_*p*_) = (*P*_*s*_ − *P*_*d*_)/[(*D*_*s*_ − *D*_*d*_)/*D*_*d*_], compliance (AC) = *π*(*D*_*s*_ × *D*_*s*_ − *D*_*d*_ × *D*_*d*_)/[4(*P*_*s*_ − *P*_*d*_)], augmentation index (AI) = Δ*P*/*P*_*P*_, pulse wave velocity deduced by *β* (PWV*β*) = $\sqrt{\left(\beta \times P_{d}/2\rho \right)}$, and pulse wave velocity deduced by WI (PWV-WI) = (*dP*/*dU*)/*ρ*; *D*_*s*_ is maximum systolic diameter, *D*_*d*_ is minimum diastolic diameter, *D*_*m*_ is mean blood vessel diameter, *D*_*D*_ is inside diameter difference (*D*_*s*_ − *D*_*d*_), *P*_*s*_ is systolic blood pressure, *P*_*d*_ is diastolic pressure, Pm is mean arterial pressure [(*P*_*s*_ + 2 × *P*_*d*_)/3], *P*_*P*_ is pulse pressure, Δ*P* is the difference between the systolic pressure wave peak and the coincident position of the outward pulse wave and the backward wave, *dP*/*dU* is the ratio of pressure change before the retracing point in the early stage of systole and flow velocity change, and *ρ* is blood density.

### 2.6. Data Analysis

Measurement data was summarized using average and standard deviation (SD) as ($\overset{-}{x}\pm s$). *t*-tests were used to test for mean differences between groups. Measurement data which do not comply with normal distribution was summarized as median *M* (25%~75%), and Mann–Whitney *U* tests were used to test for mean differences between groups. *χ* ^2^ test was applied to compare the count data, and confounding factors were adjusted via analysis of covariance. Statistical analysis was performed using SPSS 17.0 software.

## 3. Results

### 3.1. WI Waveform Difference between Renying Pulse and Cunkou Pulse

**Figure 2:**
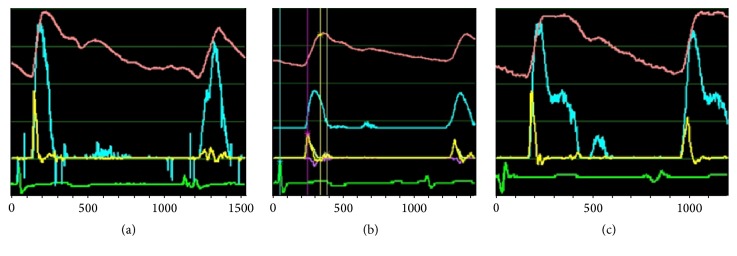
WI images of Cunkou unsmooth pulse, Cunkou normal pulse, and Renying pulse. *P* (red) is pressure wave curve, *U* (blue) is blood flow wave curve, and WI (yellow) is WI wave curve. (a) is Renying pulse (corresponding to normal pulse). (b) is Cunkou normal pulse. (c) is Cunkou unsmooth pulse. The green line refers to the electrocardiogram of the limb.

In this section, the WI waveform difference of normal pulse at carotid artery between Renying pulse and Cunkou pulse is illuminated. Carotid artery is closer to the heart compared to the Cunkou pulse and far away from peripheral resistance. The clinical wave of Renying pulse is a typical WI wave, which is formed by conspicuous amplitude of *W*1 and *W*2 and small amplitude of NA. See in Figure 2(a). While the characteristic of Cunkou normal pulse could be summarized as follows, compared to that of Renying pulse (carotid artery), its *W*1 amplitude is smaller and NA wave is more obvious, while the *W*2 wave is indistinct or even invisible, and the predejection time (*R*-1st) time is longer than that of Renying pulse (carotid artery) due to its remote distance from the heart. The *P* wave of predicrotic wavefront (also called tide wave, h3) arises earlier than that of Renying pulse, and it is closer to the principal wave (h1) because of the closer distance to peripheral resistance. The principal *U* wave of Renying pulse looks like “Λ” shape, while it looks like an arched blunt “*∩*” shape in Cunkou pulse, and the amplitude of *U* wave in Cunkou pulse is smaller with slower blood flow.  Figure 2(b) shows the Cunkou normal pulse (Figure 2(b)). There are two changing *U* wave phases in Cunkou normal *P* wave ascending phase; the former one is blood flow accelerating phase, and the latter one is decelerating phase. According to the equation, WI = (*dP*/*dt*)(*dU*/*dt*), during the accelerating phase, the product of (*dP*/*dt*)(*dU*/*dt*) is positive (forward wave), that is, *W*1, and thereafter the product of (*dP*/*dt*)(*dU*/*dt*) in decelerating phase is negative, that is, NA, while, in the descending phase of Cunkou *P* wave, there is decelerating phase appearing in blood flow, which makes the product of (*dP*/*dt*)(*dU*/*dt*) positive, that is, *W*2. Since the reflected tide wave is relatively short and the amplitude change of *P* and *U* wave is indeed insignificant, so the figure of *W*2 in Cunkou pulse is inconspicuous or even disappeared, which is shown in Figure 2. The direction of the principal *U* wave in Cunkou unsmooth pulse is up, which shows hoof boots “h” shape with high amplitude and a significant notch on declined branch, resulting in the increase and early appearance of the predicrotic wave (tide wave) or integration with the principal wave (h1).

### 3.2. WI Waveform Difference between Cunkou Normal Pulse and Unsmooth Pulse

The WI features of Cunkou normal pulse were mentioned above. The direction of the principal *U* wave in Cunkou unsmooth pulse is up, which shows hoof boots “h” shape, while the normal pulse of Cunkou looks like “*∩*” and the amplitude of unsmooth pulse blood flow is higher with an obvious notch on the descending branch; the amplitude of predicrotic wave in unsmooth pulse pressure wave (*P*) is significantly high, which could be even higher than that of principal wave (h1), resulting in early appearance of the predicrotic wave (tide wave) or integration with the principal wave (h1), which forms a wide and blunt peak and decreased amplitude of dicrotic wave (h5). The *W*1 amplitude of unsmooth pulse is relatively higher than that of normal pulse due to the greater change of (*dU*/*dt*), so the average of NA and *W*2 also increases. Owing to numerous obvious notches and fluctuations on the descending branch of blood flow wave, multiple small amplitude waves appear in the later WI according to the formula of (*dP*/*dt*)(*dU*/*dt*). (Figure 2(c)).

### 3.3. Comparison of General Clinical Characteristics in 2 Groups

There was no significant difference in diastolic blood pressure and heart rate between the 2 groups (*p* > 0.05), while the difference of age, body mass index, and systolic pressure between the 2 groups was statistically significant (*p* < 0.05). See Table 1.

### 3.4. Analysis of WI Parameters

The average of *W*1, *W*2, NA, *β*, *E*_*p*_, and PWV*β* in unsmooth pulse group is higher compared to the normal pulse group (*p* < 0.05), while the average of *R*-1st, 1st-2nd, AC, and AI is relatively lower (*p* < 0.05) (see Table 2). The analysis of covariance regarded the parameters of WI as dependent variable, while it took the BMI and age as covariant. The result of comparison between groups are as follows: *F*_*W*1_ = 78.268, *p* < 0.001; *F*_*W*2_ = 12.784, *p* = 0.001; *F*_NA_ = 1.214, *p* = 0.275; *F*_*R*-1st_ = 10.397, *p* = 0.002; *F*_1st-2nd_ = 0.681, *p* = 0.413; *F*_*β*_ = 0.006, *p* = 0.939; *F*_*E*_*p*__ = 0.013, *p* = 0.910; *F*_AC_ = 1.355, *p* = 0.249; *F*_AI_ = 1113.650, *p* < 0.001; *F*_PWV*β*_ = 0.139, *p* = 0.711; and *F*_PWV-WI_ = 0.399, *p* = 0.530, which demonstrates that after adjusting the confounding factors of BMI and age, the mean difference of *W*1, *W*2, *R*-1st, and AI in two groups was still of statistically significance (*p* < 0.05).

## 4. Discussion

The first leveraged sphygmograph instrument was invented in 1860 by Vierordt. In the early 1950s, a dozen pulse instruments were developed and produced for the purpose of research in pulse diagnosis in China. There were two types of pulse waves, namely, pressure waves and volume waves, respectively, reflecting the intravascular pressure and vascular volume cycle, which could be used in scientific research and teaching. The tasks of pulse analyzer mainly included information acquisition and signal analysis; the sensor, also known as the transducer, concentrates on converting the nonelectric physical quantity of biological information such as finger pulse or radial pulse pump power converted into measurable electrical quantity. At present, there are many kinds of sensors used to obtain pulse wave, such as light sensitive element, electrical strain gage, piezoelectric crystal, monocrystalline silicon, and polyvinylidene fluoride (PVDF) piezoelectric film. The analyzing method for pulse image is analysis in time and frequency domains and so forth [15].

Pulse condition is the most sensitive physiological information of body activities and easy to observe [16]. Unsmooth pulse, which is a common pulse of numerous clinical diseases, owns a basic characteristic of being thready, slow, and short, with rough coming and going, like scraping a piece of bamboo, which suggests the lack of essence and insufficiency of blood,* qi* stagnation and blood stasis, and food-phlegm retention [7, 17, 18]. To date, the study of pulse condition apparatus based on pressure sensor deems that [19] its main characteristics could be summarized as the pulse rate is less than 58 beats per minute, the amplitude of pressure wave is less than 1.1 mV, the pulse wave curve is flat, and the pulse image presents as a low earth fortress with a slow rising and descending speed, prolonged ascending time, and sometimes a pause. The peak of the principal wave (h1) is blunt, and the position of tide wave and dicrotic notch along with dicrotic wave (h5) is relatively higher while the figure is inconspicuous or even disappeared. Li et al. [20] collected the unsmooth pulse signal from pressure sensor and transformed the time domain to the frequency domain through Fourier transformation. Set the maximum frequency of pulse range from 0 to 40 Hz, so the ratio of the power spectrum energy and the total energy of the power spectrum below 10 Hz is defined as spectral energy ratio (*K* = *E*10/*E*40). The low frequency component of the smooth pulse, unsmooth pulse, and normal below 10 Hz occupies the main energy signal. Compared to the normal pulse, the *K* value in signal of the unsmooth pulse is higher, indicating the energy of the unsmooth pulse is more concentrated in the low frequency band to make sure the energy is mainly used for the maintenance of the fundamental frequency of the heart beat instead of spreading among the high frequency of multiple harmonics. The alternating times of the positive phase and negative phase according to the phase spectrum analysis were as follows: smooth pulse (14 times) > unsmooth pulse (12 times) > normal pulse (4 times), while the interval time between alternation was just the opposite. Among the three pulses, the difference between the positive and negative phase difference of unsmooth pulse is the smallest, while the smooth pulse is the biggest. The abnormal changes of positive and negative phase spectrum between the unsmooth pulse and smooth pulse illustrate the compensatory mechanism of hemodynamics and vascular elasticity modulus and so forth. The features of unsmooth pulse collected by baroreceptors were verified in our experiments using WI technology.

The objectivity and repeatability of pulse images still remains insufficient as a result of signal instability, since the pulse condition apparatus is susceptible to the rigidity and elasticity of skin-soft tissue-radial arterial canal deformation and artificial tension [21], while the WI pulse image is almost free from outside influence since it is based on the theory of the linear relationship between the diameter change and the pressure change [22], and pressure change is measured by metering the diameter change; thus the pulse images could be more intuitionistic, recognizable, and objective. Besides, the WI pulse image is more comprehensive than the current two-dimensional pulse condition instrument since it is able to reflect the 3D dynamic details of the blood flow velocity and pressure, as well as the motion of vascular wall, which is more in line with the TCM of the location, number, shape, and power in traditional pulse conditions. Therefore, the application of WI technology in research is more scientific and objective, which serves as an accurate, noninvasive, convenient approach for clinical operation and application.

Renying pulse belongs to the elastic blood vessels and is close to the heart, away from the peripheral resistance. Therefore, its flexibility and expansion is much better. The pressure on the vascular walls generated by every ejection of cardiac cycle and velocity of blood flow were transferred to the peripheral blood vessels, plus the reflected waves, forming the intersection of pressure wave phase and velocity wave phase, forming the characteristic waveform WI.

The pressure wave of the radial artery consists of 3 components which is an incident wave generated by the blood flow and two reflected waves which are returned from the hand and the lower part of body, respectively. As the elastic arteries become stiff, PWV increases and the reflected wave from the lower body returns to the radial artery early, so that the first reflected wave is closer to principal wave (h1) and so that the AI of radial artery is more closely related to the elasticity of large arteries [23]. According to the definition of (*dP*/*dt*)(*dU*/*dt*), the *W*1 amplitude of Cunkou pulse is smaller resulting from the fact that it is closer to the peripheral resistance along with less change in blood flow compared to the Renying pulse. The *W*2 wave is inconspicuous or even disappeared since the stroke volume that arrived at Cunkou pulse is relatively less than that of the carotid artery and therefore the pressure against the vascular wall is reduced. The peripheral resistance increases, so NA is obvious, while the *R*-1st time is longer than that of Renying pulse since the Cunkou is far away from the heart. Due to the relative far distance between the Cunkou and heart, stronger peripheral resistance is apparent, with vascular wall full of smooth muscle and poor vascular elasticity, so the velocity of pulse wave is much faster, resulting in the reflected wave appearing before the systole, which extends the systole, so the predicrotic wave of pressure wave appears earlier than that of Renying pulse and much closer to the principal wave (h1). Because of the relative far distance between the Cunkou and heart, the blood flow volume and power of radial artery is smaller than Renying pulse; therefore the blood flow slows and the amplitude of blood flow is smaller, presenting as “*∩*” shape.

Unsmooth pulse can be classified as weak pulse without power, obstructed pulse, and potent pulse. The first two types were excluded from our study. Since unsmooth pulse is thready and indicates blood insufficiency or stasis, along with the stiffness of elastic artery, and the increase of tension and pulse wave velocity (PWV), the early reach at Cunkou of reflected wave from the lower part of body making the reflected wave of the first wave move towards the principal wave (h1) besides the velocity of blood flow was inconsistent (*dU*/*dt*), with the large variation of flow velocity leading to the increase amplitude of *W*1, NA, and *W*2. Owing to numerous obvious notches and fluctuations on the descending branch of blood flow wave, multiple small amplitude waves appear after the WI according to the formula of (*dP*/*dt*)(*dU*/*dt*).

Unsmooth pulse often results from arteriosclerosis, high blood pressure, hyperlipidemia, and so forth. So the peripheral resistance and PWV accelerates and postaortic load increases, resulting in the increase and early appearance of the predicrotic wave (tide wave) or integration with the principal wave (h1). Since dicrotic wave (h5) is a potent indicator of the compliance of large vessels [19], so it shows that the amplitude of dicrotic wave (h5) decreases. The high amplitude of unsmooth pulse with an obvious notch on the descending branch makes the curve look like a hoof boot “h” type. Its formation is correlated with the obstructed, highly resistant, and inconsistent velocity of blood flow of unsmooth pulse.

After detecting the distensibility and compliance of Cunkou pulse via WI technique, we found that unsmooth pulse indicated the vascular sclerosis with the increase of PWV*β*. In order to ensure the blood supply for the other organs, the output of the heart can be increased by feedback, so that the heart rate is accelerated, and the preparation time of the heart is shortened, so that there is decrease of *R*-1st, 1st-2nd. *β*, *E*_*p*_, and AC are important indicators of arteriosclerosis; the ascending of *β* and *E*_*p*_ in unsmooth pulse and the descending of AC conform with the* qi* stagnation and blood stasis of unsmooth pulse or other related diseases. Our study also illustrates that when the AI of unsmooth pulse decreases, which predicts the arteriosclerosis, the systolic blood pressure rises and the pulse pressure (*P*_*P*_) ascends, while the vascular elasticity of unsmooth pulse decreases.

And the acceleration of PWV makes the coincident site of outward pulse wave and backward wave in advance and the amplitude increase, reducing the gap between it and the highest point of the principal systolic wave (h1); that is, Δ*P* decreases. And if the augmentation index (AI) equals Δ*P*/*P*_*P*_, then the AI of unsmooth pulse is lower than that of normal pulse. If AI equals *P*2/*P*1, then the AI of unsmooth pulse is higher than that of normal pulse. As for the clinical application of AI, there are numerous researches on how to estimate the central aortic pressure (CAP) via the AI of Cunkou pulse. There are basically two ways to measure central aortic pressure, namely, direct method and indirect method. The direct pressure measurement was an invasive method and usually left ventricular catheter is applied to measure the ascending aortic pressure. However, in recent years, study found that there is a close relationship between the pressure waveform of peripheral muscular artery (radial artery) and that of elastic artery (aorta), and a mathematical conversion relationship between the two can be established [8]. According to the above principle, a noninvasive pulse wave detector has been invented to detect the central aortic pressure and augmentation index (AI). The central hemodynamic measurements employed in this study have been previously validated [9, 10]. Radial artery waveforms were obtained with a high-fidelity micromanometer (SPC-301; Millar Instruments, Houston, TX, USA) from the wrist and a corresponding central waveform was generated with a generalized transfer function (SphygmoCor, AtCor Medical, Sydney, Australia), which has been widely authorized by using invasive measurements of radial waveforms [9, 10, 24]. The correlation and repeatability were both favorable. The AI of central artery and arterial stiffness were closely related to the risk of future major adverse cardiovascular events (MACE), which makes AI become an essential indicator for screening high-risk groups, and optimizing the antihypertensive program [25, 26]. The noninvasive device for measuring central aortic blood pressure and arterial elasticity function has a bright prospect in clinical application. At present, the idea of using these methods for clinical practice guidance is gradually established, with the research data to enrich, of which some may become a useful indicator of cardiovascular risk stratification and therapeutic drug monitoring in the next few years. From the data above, it is obvious that the theory of wave intensity (WI) still exhibits crucial clinical significance, and we are delighted to see its further application at present.

Pulse diagnosis is one of the indispensable ways of treatment in Traditional Chinese Medicine, which has always been highly valued by generations of physicians and plays a significant historical role. There are different forms of unsmooth pulse; thus study of varied categories of unsmooth pulse is still required to expand and to further consummate the construction of pulse condition images of unsmooth pulse. In the current era, it is expected to find a way to link up Eastern and Western medicine with the advanced technology.

## 5. Conclusions

The application of WI (WI = (*dP*/*dt*)(*dU*/*dt*)) can be used as a new pulse concept for the further direction of research in this field. The application of WI technology in pulse makes the image of unsmooth pulse more intuitionistic, recognizable, and objective, which is conducive to the succession of Chinese traditional sphygmology.

## Figures and Tables

**Table 1:** Comparison of general characteristics between normal pulse and unsmooth pulse ($\overset{-}{x}\pm s$).

Item	Normal pulse [27]	Unsmooth pulse	*t*	*p*
Age (y)	33.13 ± 7.67	62.00 ± 4.95	−17.317	<0.001
BMI (kg/m^2^)	21.56 ± 1.23	22.93 ± 2.04	−3.143	0.003
SBP (mmHg)	125.60 ± 5.67	133.33 ± 14.02	−2.801	0.008
DBP (mmHg)	75.00 ± 2.39	73.47 ± 8.58	0.943	0.353
HR (b/min)	68.40 ± 7.10	68.90 ± 6.49	−0.285	0.777

**Table 2:** Comparison of WI parameters between normal pulse and unsmooth pulse ($\overset{-}{x}\pm s$) or *M* (25%~75%).

Item	Normal pulse [27]	Unsmooth pulse	*T* or *Z* value	*p*
W1 (×10^3^ mmHg·m/s^3^)	7.64 ± 1.19	25.50 ± 4.01	−23.361	<0.001
W2 (×10^3^ mmHg·m/s^3^)	0.12 ± 0.16	3.16 ± 1.56	−10.589	<0.001
NA (mmHg·m/s^2^)	30.58 (24.76~34.72)	50.24 (33.73~66.92)	−4.746^*∗*^	<0.001
R-1st (ms)	206.50 (204.00~245.75)	140.00 (133.00~176.25)	−6.664^*∗*^	<0.001
1st-2nd (ms)	326.50 (156.50~363.00)	236.00 (204.25~266.00)	−2.619^*∗*^	0.009
*β*	25.69 ± 12.17	33.71 ± 10.58	−2.724	0.009
*E*_*p*_ (kPa)	332.50 (137.00~513.00)	479.00 (435.25~514.50)	−2.159^*∗*^	0.031
AC (mm^2^/kPa)	0.03 (0.01~0.08)	0.02 (0.02~0.03)	−0.389^*∗*^	0.697
AI (%)	92.19 ± 5.03	1.40 ± 3.50	81.140	<0.001
PWV*β* (m/s)	11.00 (7.20~13.50)	13.05 (12.35~13.60)	−2.279^*∗*^	0.023
PWV-WI (m/s)	4.48 (4.32~4.48)	4.98 (3.52~6.06)	−0.761^*∗*^	0.447

*∗* represents *Z* value.

## Abbreviations

WI: Wave intensity
ET: Echo tracking
*P*: Pressure
*U*: Velocity
SD: Standard deviation
BMI: Body mass index
h1: Principal wave
h3: Predicrotic wave (tide wave)
h5: Dicrotic wave
1st-2nd: Ejection period
*R*-1st: Predejection
*W*1: WI of forward traveling compression wave
*W*2: WI of forward traveling expansion wave
NA: Negative area of WI wave
*β*: Vascular Stiffness
*E*_*p*_: Elastic modulus
AC: Compliance
PWV*β*: Pulse wave velocity deduced by *β*
PWV-WI: Pulse wave velocity deduced by WI
AI: Augmentation index
TCM: Traditional Chinese Medicine.
